# Anal canal adenocarcinoma with neuroendocrine features accompanying secondary extramammary Paget disease, successfully treated with modified FOLFOX6: a case report

**DOI:** 10.1186/s12885-018-5084-0

**Published:** 2018-11-20

**Authors:** Masamichi Yamaura, Takeshi Yamada, Rei Watanabe, Hitomi Kawai, Suguru Hirose, Hiroki Tajima, Masashi Sato, Yuichi Uchida, Daisuke Suganuma, Yoshiyuki Yamamoto, Toshikazu Moriwaki, Ichinosuke Hyodo

**Affiliations:** 1 Department of Gastroenterology, University of Tsukuba Hospital, 1-1-1 Tennodai, Tsukuba, Ibaraki, 305-8575 Japan; 2 Division of Dermatology, University of Tsukuba Hospital, Ibaraki, Japan; 3 Division of Pathology, University of Tsukuba Hospital, Ibaraki, Japan

**Keywords:** Anal canal cancer, Adenocarcinoma with neuroendocrine features, Extramammary Paget’s disease, mFOLFOX6

## Abstract

**Background:**

Anal canal cancer occasionally accompanies extramammary Paget disease. Although most of them are squamous cell carcinoma, anal canal adenocarcinoma with neuroendocrine features accompanying secondary extramammary Paget disease has never been reported.

**Case presentation:**

Here, we report a 76-year-old man presented with pruritus in the perianal area. Investigation revealed a fist-sized perianal erythema, diffuse liver tumors, and right inguinal lymph node swelling. Pathological examination of biopsies from the erythema suggested secondary extramammary Paget disease with positive cytokeratin-7 and -20 expressions and negative GCDFP-15 expression. The anal canal tumor was confirmed by digital examination and endoscopy. Biopsies from the anal canal tumor, swollen lymph node, and Paget lesion all showed poorly differentiated adenocarcinoma with neuroendocrine features expressing synaptophysin and chromogranin A. Serum CEA and NSE levels were high, 809.4 ng/ml and 85.8 ng/ml, respectively. After chemotherapy with modified FOLFOX6 for 2 months, the Paget lesion disappeared, and the primary anal canal tumor and liver metastases shrunk remarkably. Serum CEA and NSE levels decreased promptly to within normal ranges.

**Conclusions:**

This is a clinically significant case, as it reveals novel pathological features about anal canal cancer with secondary Paget disease and successfully treated with modified FOLFOX6. Careful pathological investigation and appropriate treatment choice are needed for this rare cancer.

## Background

Anal canal cancer is uncommon [1, 2], and approximately 90% of cases are associated with human papillomavirus (HPV) infection [3]. The incidence of anal canal cancer has gradually increased over the last few decades [4]. Histological findings are usually squamous cell carcinoma and the remaining including adenocarcinoma, neuroendocrine carcinoma (NEC), melanoma, lymphoma, undifferentiated carcinoma, and mesenchymal tumors [5, 6]. Anal canal adenocarcinoma is less associated with HPV infection [7] and divided into two subtypes according to the presence of mutations in EGFR signaling pathway and expression of the immune checkpoint molecules [8].

Anal canal cancer is known to occasionally accompany with secondary extramammary Paget disease (EPD) [2, 9], which is characterized histologically as the intraepidermal proliferation of unique tumor cells (Paget cells) found in classic mammary Paget disease. Although both primary and secondary EPD show similar erosive erythematous plaque, the prognosis is different between these two EPD and accurate differential diagnosis is important [10].

Here we present a rare case of adenocarcinoma with neuroendocrine features of the anal canal accompanying secondary EPD with diffuse liver involvements, successfully treated with an oxaliplatin-containing regimen.

## Case presentation

**Fig. 1:**
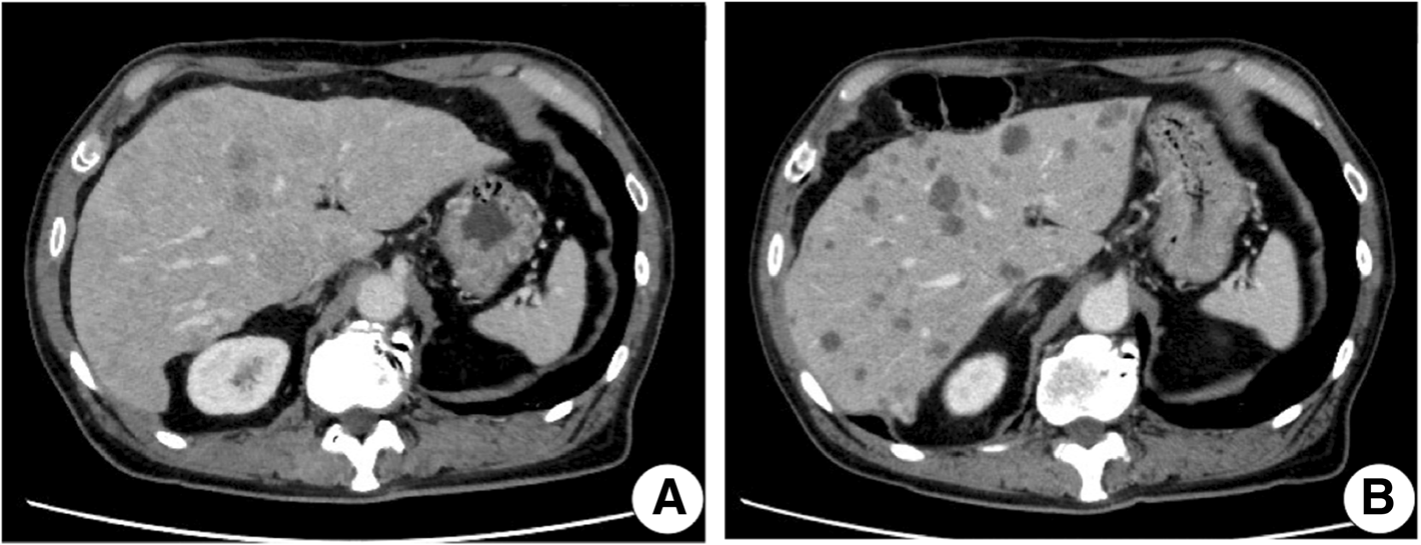
CT scan showed diffuse liver metastases before treatment (**a**); cystic morphologically changed after 4 courses of mFOLFOX6 (**b**)

**Fig. 2:**
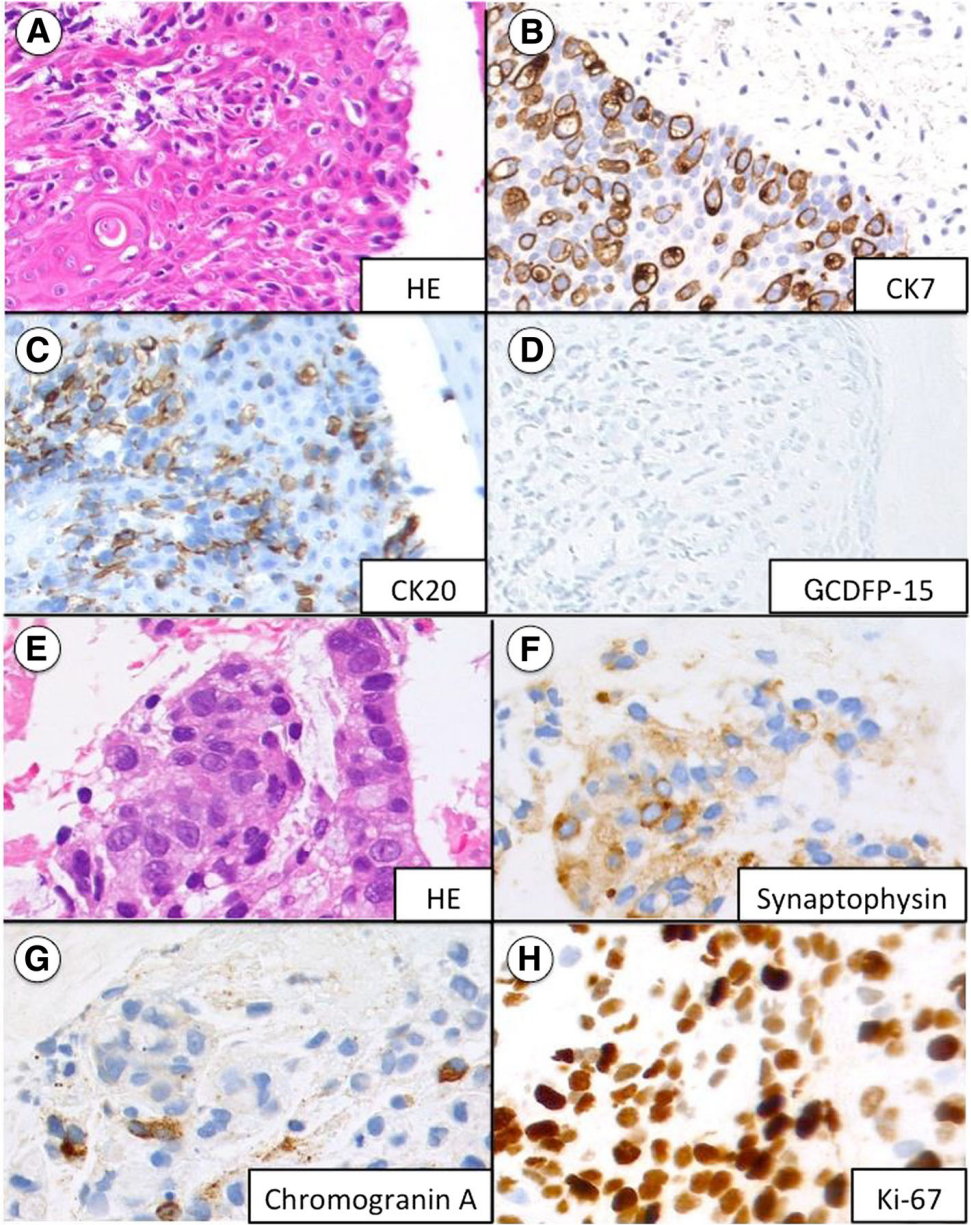
Pathological examination of extramammary Paget disease (**a**, **b**, **c**, **d**) and primary lesion of anal canal (**e**, **f**, **g**, **h**) revealed poorly differentiated adenocarcinoma with neuroendocrine features. (HE: hematoxylin eosin staining, CK7/CK20: Cytokeratin7/20, GCDFP-15: gross cystic disease fluid protein-15)

A 76-year-old man with a history of hypertension and benign prostatic hyperplasia consulted a dermatologist with a complaint of pruritus in the perianal area. The doctor diagnosed this area as eczema, and had prescribed Corticosteroid ointment for him for 10 months. Because his symptom did not improve, he received a colonoscopy to check for colorectal malignancy. However, no anal canal lesion was noticed at that time. Two months later, multiple liver lesions were incidentally found during follow-up ultrasonography for his prostatic hyperplasia. Computed tomography (CT) scan revealed multiple liver lesions (Fig. 1a) and right inguinal lymph node swelling. Pathological examination of biopsies obtained from the perianal erythema showed infiltrating Pagetoid cells and poorly differentiated adenocarcinoma (Fig. 2a). Immunohistochemistry (IHC) demonstrated malignant cells positive for cytokeratin (CK)-7 and − 20 (Fig. 2b and c) and negative for gross cystic disease fluid protein-15 (GCDFP-15) (Fig. 2d). These findings suggested secondary EPD. The lymph node was also pathologically diagnosed as a metastasis. He was referred to our hospital for further examination and treatment.

**Fig. 3:**
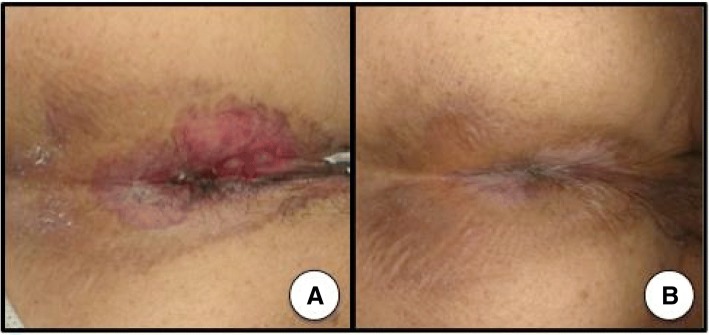
Paget disease was seen in perianal region before treatment (**a**) and disappeared after 4 courses of mFOLFOX6 (**b**)

**Fig. 4:**
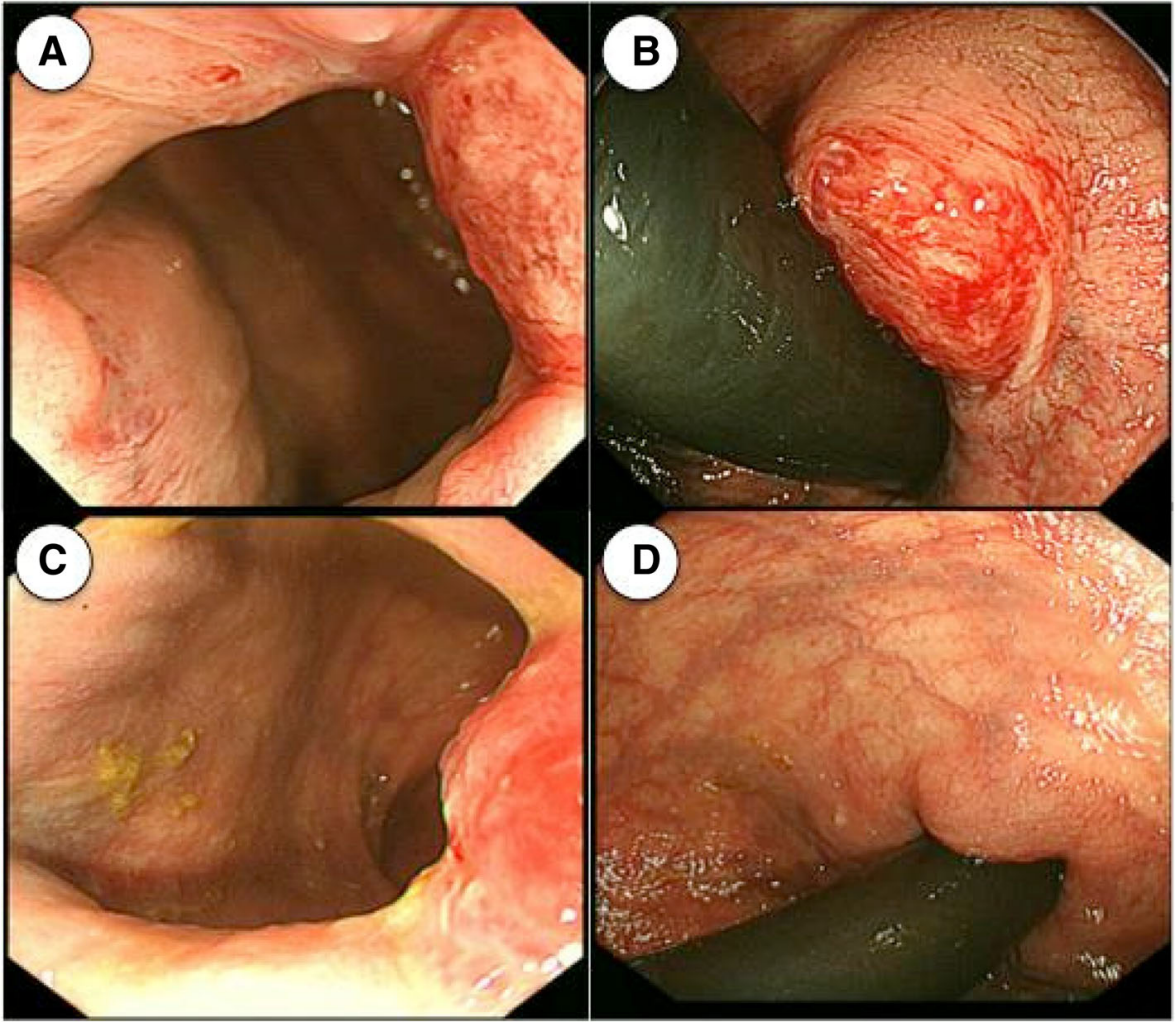
Endoscopy revealed the primary anal canal lesion like a submucosal tumor (**a** and **b**), which shrunk after 4 course of mFOLFOX6 (**c** and **d**)

On admission, his European Cooperative Oncology Group performance status was 0. Physical examination revealed hepatomegaly and erythematous perianal skin lesion (Fig. 3a). Elastic hard tumor in the anal canal was palpable by digital examination. Serum carcinoembryonic antigen (CEA), neuron specific γ- enolase (NSE), and lactate dehydrogenase (LDH) levels were high, with 809.4 ng/mL (normal range, 0 to 5 ng/mL), 85.8 ng/mL (normal range, 0 to 16.3 ng/mL), and 1176 U/L (normal range, 115 to 245 U/L), respectively. Carbohydrate antigen 19–9 level was normal. Endoscopy showed an elevated tumor of the anal canal like a submucosal tumor (Fig. 4a and b). Pathological examination revealed poorly differentiated adenocarcinoma (Fig. 2e) with neuroendocrine features of positive synaptophysin and chromogranin-A expressions (Fig. 2f and g). Ki-67 showed a high proliferation index of 60% (Fig. 2h). A *KRAS* mutation at codon 12 was detected in the primary anal canal lesion. The previously diagnosed perianal skin lesion and lymph node tumor showed the same pathological features. Finally, the patient was diagnosed with metastatic anal canal adenocarcinoma with neuroendocrine features, accompanying secondary EPD.

**Fig. 5:**
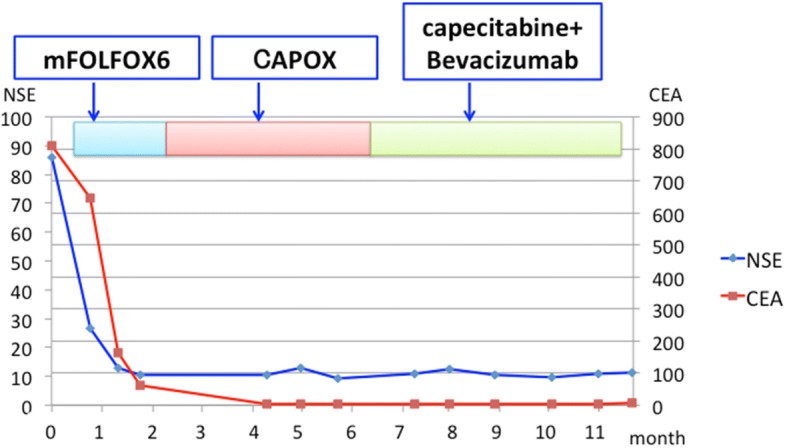
Patient’s clinical course. Remarkable tumor shrinkage and normalization of serum CEA and NSE levels continue for more than 11 months. (CEA, serum carcinoembryonic antigen; NSE, serum neuron specific enolase)

He received chemotherapy with mFOLFOX6 (oxaliplatin 85 mg/m^2^, bolus 5-FU 400 mg/m^2^, and folinic acid 200 mg/m^2^ on day 1 with 46-h infusional 5-FU 2400 mg/m^2^, every 2 weeks). Soon after treatment, his hepatomegaly improved day by day. CT scan after 4 courses of mFOLFOX6 showed remarkable tumor shrinkage and morphological changes to homogenous nonenhanced lesions (Fig. 1b), and the EPD disappeared (Fig. 3b). Serum levels of LDH, CEA, and NSE decreased promptly to within normal range (Fig. 5). The primary anal canal lesion also responded to the treatment (Fig. 4c and d). The treatment regimen of mFOLFOX6 was switched to CAPOX (capecitabine 2000 mg/m^2^/day for 14 days and oxaliplatin 130 mg/m^2^ on day 1, every 3 weeks) due to thrombus formation around the central venous catheter. Currently, he is receiving capecitabine plus bevacizumab together with edoxaban to prevent secondary deep venous thrombosis after removal of central venous catheter as a maintenance therapy, and a good partial response with normal serum tumor markers has been maintained for more than 11 months after the initial treatment.

## Discussion

Anal canal cancer is occasionally associated with secondary EPD. Goldman et al. reported the frequency of secondary EPD accompanied with anal canal cancer as 33% [11]. The most common histological findings with secondary EPD are adenocarcinoma and squamous cell carcinoma [12]. Other histological types with secondary EPD are rare and there are only a few case reports including NEC [13], signet ring cell carcinoma [14], and mucinous carcinoma [15, 16]. The present case was extremely rare in terms of the particular histological type accompanied with secondary EPD.

It is not easy to distinguish between secondary and primary EPD by the clinical and histological findings, especially if primary EPD invades the epidermis or if an underlying visceral carcinoma is not apparent. IHC with CK-7, CK-20, and GCDFP-15 may be useful to distinguish them. In secondary EPD, the tumor cells are positive for CK7 and CK20, but negative for GCDFP-15, whereas primary EPD is commonly positive for GCDFP-15 and CK-7, but negative for CK-20 [17–21]. A detailed examination to detect primary tumors should be performed especially in anorectal lesions such as the present case [10].

In this case, anal canal lesion was missed by previous colonoscopy. We think the reason for difficulty in detection is based on the feature that the anal lesion developed like submucosal tumor. We could detect the lesion because we had information about secondary Paget disease and strongly suspected the anal cancer by digital examination. Most anal canal squamous cell carcinoma is caused by high risk HPV, anal canal adenocarcinoma is less related with HPV [7]. This patient did not have certain sexual history and had KRAS-mutant tumor. Although we did not evaluate HPV infection in this case, the carcinogenesis of this patient seemed to be less associated with HPV infection [8].

There is no report of mixed adenoneuroendocrine carcinoma of anal canal cancer, and only one case, involving an elderly female patient, of NEC with squamous intraepithelial neoplasm of the anal canal has been reported [22]. The present case was a rare anal canal adenocarcinoma with neuroendocrine features, in which prognosis seemed poor with metastases like those reported in intestinal NEC [23, 24].

Fortunately, the patient was successfully treated with mFOLFOX6, which is a standard treatment for colorectal adenocarcinoma. CT scan after 4 courses of mFOLFOX6 showed remarkable tumor shrinkage and morphological changes to homogenous nonenhanced lesions. CT-based morphological changes correlate with pathologic response and overall survival among patients with colorectal liver metastases treated with bevacizumab-containing chemotherapy [25]. NEC is commonly treated with a platinum-based doublet, such as etoposide plus cisplatin, following the treatment guideline for small-cell lung cancer. We selected mFOLFOX6 regimen for the patient because the lesion included both components of adenocarcinoma and NEC. Baba, et al. reported a case of anal canal NEC successfully treated with mFOLFOX6 [26]. Further studies are needed to develop the optimal treatment for these particular types of cancer.

## Conclusions

We reported here the rare case of anal canal adenocarcinoma with neuroendocrine features accompanying secondary EPD, and found mFOLFOX6 to be a very effective treatment. Careful evaluation is necessary for perianal skin lesion as anal canal cancer with EPD might be hidden.

## Abbreviations

CEA: Carcinoembryonic antigen
CK-20: Cytokeratine-20
CK-7: Cytokeratine-7
CT: Computed tomography
EPD: Extramammary Paget disease
FDG PET: ^8^F-fluorodeoxy glucose positron emission tomography
GCDFP-15: Gross cystic disease fluid protein-15
HPV: Human papillomavirus
LDH: Lactate dehydrogenase
mFOLFOX6: modified FOLFOX6 regimen
NEC: Neuroendocrine carcinoma
NSE: Neuron specific γ- enolase
